# A *Salmonella* Typhimurium-Typhi Genomic Chimera: A Model to Study Vi Polysaccharide Capsule Function In Vivo

**DOI:** 10.1371/journal.ppat.1002131

**Published:** 2011-07-28

**Authors:** Angela M. Jansen, Lindsay J. Hall, Simon Clare, David Goulding, Kathryn E. Holt, Andrew J. Grant, Piero Mastroeni, Gordon Dougan, Robert A. Kingsley

**Affiliations:** 1 The Wellcome Trust Sanger Institute, The Wellcome Trust Genome Campus, Hinxton, Cambridge, United Kingdom; 2 Department of Veterinary Medicine, University of Cambridge, Cambridge, United Kingdom; Stanford University School of Medicine, United States of America

## Abstract

The Vi capsular polysaccharide is a virulence-associated factor expressed by *Salmonella enterica* serotype Typhi but absent from virtually all other *Salmonella* serotypes. In order to study this determinant *in vivo*, we characterised a Vi-positive *S.* Typhimurium (C5.507 Vi^+^), harbouring the *Salmonella* pathogenicity island (SPI)-7, which encodes the Vi locus. *S.* Typhimurium C5.507 Vi^+^ colonised and persisted in mice at similar levels compared to the parent strain, *S.* Typhimurium C5. However, the innate immune response to infection with C5.507 Vi^+^ and SGB1, an isogenic derivative not expressing Vi, differed markedly. Infection with C5.507 Vi^+^ resulted in a significant reduction in cellular trafficking of innate immune cells, including PMN and NK cells, compared to SGB1 Vi^−^ infected animals. C5.507 Vi^+^ infection stimulated reduced numbers of TNF-α, MIP-2 and perforin producing cells compared to SGB1 Vi^−^. The modulating effect associated with Vi was not observed in MyD88^−/−^ and was reduced in TLR4^−/−^ mice. The presence of the Vi capsule also correlated with induction of the anti-inflammatory cytokine IL-10 in vivo, a factor that impacted on chemotaxis and the activation of immune cells in vitro.

## Author Summary

Pathogens of the genus *Salmonella* are closely related yet cause distinct diseases and have different host-range. *Salmonella* Typhi causes a systemic disease called typhoid fever specifically in humans, and is commonly modelled using a surrogate host-pathogen combination, namely *Salmonella* Typhimurium infection in mice. However, key virulence mechanisms of *S.* Typhi depend on the Vi polysaccharide capsule that is not expressed by *S.* Typhimurium. In order to study the function of the Vi capsule we characterised a *S.* Typhimurium/*S.* Typhi chimera that expresses the Vi polysaccharide in a regulated manner similar to that previously described in *S.* Typhi. The impact of Vi expression on immune cell populations in the spleen and mesenteric lymph nodes, and the pattern of intracellular cytokine response was determined 24 hours after i.v or i.g inoculation. Infection of mice with *S.* Typhimurium expressing Vi polysaccharide resulted in a blunted response in recruitment of NK and PMN cells. This was reflected in a blunted proinflammatory cytokine response, but a striking increase in the anti-inflammatory cytokine IL-10. IL-10 was expressed in macrophage, dendritic cells and NK cells in the mouse spleen, specifically in response to infection with *S.* Typhimurium expressing Vi polysaccharide. Indeed, neutralisation of this IL-10 production lead to increased migration and activation of splenocytes *in vitro*. This model can be used to develop Vi based vaccines as well as to study the impact of Vi expression on pathogenesis.

## Introduction

The genus *Salmonella* comprises serotypes with a range of host adaptation, and spectrum of disease syndromes ranging from self-limiting gastroenteritis, bacteraemia and typhoid fever. The outcome of the host-pathogen interaction is dependent on the combination of the host species, host immune status and the repertoire of virulence factors encoded in the genome of the *Salmonella* pathogen. Typhoid fever is a systemic disease caused by *Salmonella enterica* serovar Typhi (*S.* Typhi), a serotype that is highly host-adapted to the human host. Typhoid disease is characterised by a slow onset, protracted fever and a relatively high frequency of chronic carriage [1]. Although fever is ultimately an important feature of typhoid, progression of the disease is relatively slow and septic shock is uncommon. Although pyrogenic cytokines are elevated in typhoid patients [2], [3], they are nonetheless low relative to patients with sepsis [4], [5].

Typhoid fever has been extensively studied using the surrogate pathogen *S.* Typhimurium infections in genetically susceptible mouse. This model has been used successfully to study many aspects of typhoid fever where *S.* Typhi and *S.* Typhimurium employ common virulence mechanisms. A significant antigenic difference between *S.* Typhi and *S.* Typhimurium is the expression of the Vi polysaccharide capsule by Typhi. The Vi locus is encoded on the 134 kb *Salmonella* pathogenicity island (SPI) 7 that is not present in non-typhoid *Salmonella* serotypes such as *S.* Typhimurium. The Vi locus, known as *viaB*, encodes genes involved in Vi regulation (*tviA*), biosynthesis (*tviBCDE*) and export (*vexABCDE*) [6]. *S.* Typhi that express Vi are more virulent than equivalent Vi-negative *S.* Typhi in volunteers and Vi is expressed by virtually all clinical isolates of *S.* Typhi [7]. TNF-α production by J774 macrophage-like cells and transcription of GRO-a and IL-17 genes in the intestine of streptomycin pre-treated mice, bovine ileal loops and human colonic explants was decreased as a result of expression of the Vi polysaccharide by *S.* Typhimurium [8], [9]. Furthermore, TNF-α and i-NOS expression in the liver of mice was similarly decreased in response to expression of Vi [10].

Here we characterise the expression of the Vi polysaccharide capsule by a *S.* Typhimurium/*S.* Typhi genomic chimera in vitro, and the early innate immune response to infection in the murine typhoid model. We test the hypothesis that *S.* Typhimurium containing the entire SPI-7 region and expressing the Vi polysaccharide capsule modulates the murine immune response during the systemic phase of infection resulting in altered immune cell populations in the spleen and mesenteric lymph nodes and the intracellular cytokine response. Our results further define the genetic basis of *S.* Typhi pathogenesis and host adaptation, and propose an improved murine typhoid model for developing intervention strategies to combat typhoid fever, including Vi polysaccharide based vaccines.

## Results

### *S.* Typhimurium C5507 Vi^+^ harbours SPI-7 of *S.* Typhi and expresses Vi capsular polysaccharide

**Figure 1:**
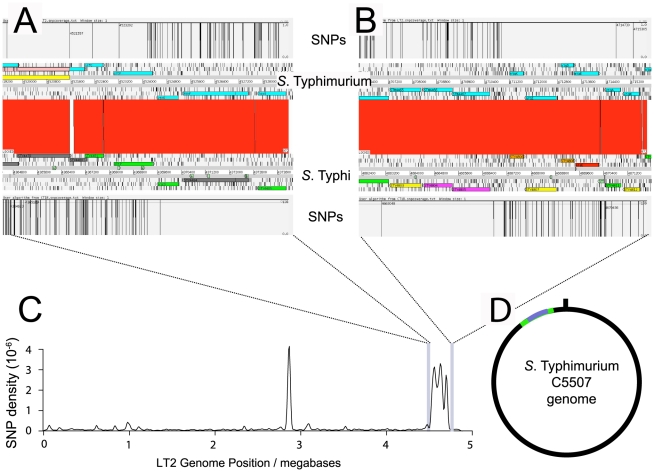
SNP density of *S.* Typhimurium C5.507 Vi^+^ mapped to *S.* Typhimurium LT2 and *S.* Typhi CT18. The boundaries of genomic sequence of *S.* Typhimurium and *S.* Typhi origin were determined by calculating the SNP density identified by mapping Illumina GA short reads from *S.* Typhimurium C5507 to the *S.* Typhimurium LT2 and *S.* Typhi Ty2 reference genome sequence. Artemis comparison tool (ACT) view of the left (A) and right (B) boundaries are shown with the position of SNPs in *S.* Typhimurium C5507 sequence reads mapped to either the *S.* Typhimurium LT2 or *S.* Typhi Ty2 genome. The SNP density (C) determined by mapping Illumina GA short reads from *S.* Typhimurium C5507 to the *S.* Typhimurium LT2 reference genome. The peak at 2.8 megabases is due to related but distinct prophage elements in *S.* Typhimurium C5507 genome relative to LT2 and the peak at 4.5 to 4.8 Mb is the *S.* Typhi genome sequence flanking SPI-7. Sequence reads from SPI-7 were not mapped to LT2 since this region is absent from *S.* Typhimurium. The deduced mosaic structure of the *S.* Typhimurium C5507 genome (D) with genomic sequence of *S.* Typhimurium C5 origin (black line) and *S.* Typhi Ty2 genome (green line) and SPI-7 (blue line) indicated.

*S.* Typhimurium C5.507 Vi^+^ was constructed by hfr conjugation between *S.* Typhi Ty2 and *S.* Typhimurium C5 during which a previously undefined region of the chromosome of *S.* Typhi Ty2 was transferred to *S.* Typhimurium (Personal Communication, M.Y. Poppoff). An exconjugant designated C5.507 Vi^+^, was agglutinated with anti-Vi antiserum and antiserum raised to the somatic antigens O4, O5 and O12. This suggested that genes required for Vi biosynthesis are present in C5.507 Vi^+^, but that the *S.* Typhi-derived genome did not include genes encoding the determinants of *S.* Typhi O antigens (O9, O12). To define the extent of *S.* Typhi genome we used an Illumina Genome Analyzer (Illumina, GA) to determine 36 bp single end nucleotide reads from 300 bp fragments of C5.507 Vi^+^ genome. We then determined *S.* Typhi and *S.* Typhimurium-specific single nucleotide polymorphisms (SNPs) by mapping reads to the complete genome sequences of *S.* Typhimurium LT2 and *S.* Typhi CT18 (Figure 1). As these were single end reads we were unable to assemble the genome, but nonetheless the *S.* Typhimurium and *S.* Typhi SNP density defined the origin of the chimeric genome sequence (Figure 1). Most of the uniquely mapped reads of C5.507 Vi^+^ contained a low SNP frequency when mapped to the LT2 genome and a relatively high SNP frequency when mapped to *S.* Typhi CT18 consistent with a relatively small region of the *S.* Typhi genome recombining into a predominantly *S.* Typhimurium C5 background. Two regions of elevated SNP frequency were identified, when Illumina reads were mapped to the LT2 genome. The first mapped to the Fels-2 prophage element of LT2, showing that a related but distinct phage is present in the C5.507 Vi^+^ genome reflecting the distinct phage repertoire of LT2 and C5. A second region of high SNP density mapped to the SPI-7 region of *S.* Typhi. This spanned the region from the 5′ end of the *gltP* gene to the intergenic region of STY4805 and STY4806, a total of 298 kb of the *S.* Typhi genome including the entire SPI-7 genomic island. This indicated that nearly 7% of the *S.* Typhi genome was present in the C5.507 Vi^+^.

**Figure 2:**
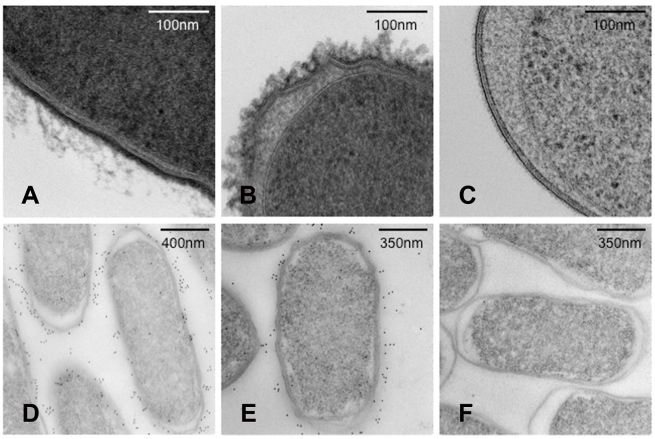
Transmission electron microscopy (TEM) images of *S.* Typhi and *S.* Typhimurium showing expression of Vi polysaccharide. S. Typhi (A), S. Typhimurium C5507 (B), *S.* Typhimurium SGB1 (C5507 D*tviB*::kan^r^) (C) visualised using TEM. S. Typhi (D), S. Typhimurium C5507 (E), *S.* Typhimurium SGB1 (C5507 D*tviB*::kan^r^) (F) visualised using TEM in conjunction with immunogold labelling using anti-Vi antibody.

**Figure 3:**
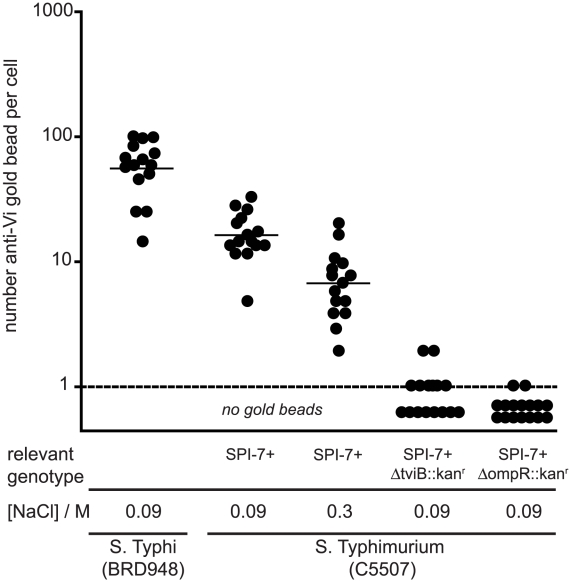
Enumeration of anti-Vi antibody immuno-gold labelling of *S.* Typhi and *S.* Typhimurium. The number (filled circle) and the mean (horizontal bar) of anti-Vi antibody coated gold particles associated with *S.* Typhi BRD948, and *S.* Typhimurium C5507 either with no additional mutations or in which the *tviB* or *ompR* gene were deleted. Bacteria were cultured in medium containing NaCl of either 0.09 M (low osmolarity) or 0.3 M (high osmolarity).

The *viaB* locus harbours genes necessary for the biosynthesis, secretion and anchoring of the Vi polysaccharide antigen on the bacterial cell surface [6]. Surface structures resembling a capsule were visualised by transmission electron microscopy (TEM) of the control *S.* Typhi BRD948 and *S.* Typhimurium C5.507 Vi^+^ cultured in rich medium containing 0.09 M NaCl. This structure was absent from *S.* Typhimurium C5.507 Vi^+^ in which the *tviB* gene, that encodes an essential component of the biosynthesis pathway, had been deleted (SGB1) (Figures 2A–C). Furthermore, SGB1 did not agglutinate with anti-Vi antiserum. The presence of Vi on the surface of *S.* Typhi and *S.* Typhimurium was visualised and semi-quantified by immunogold labelling with anti-Vi coated gold beads (Figures 2D–E and Figure 3). *S.* Typhimurium SGB1 cells were not associated with gold beads, while in contrast C5.507 Vi^+^ were significantly associated with anti-Vi^+^ coated gold beads. In *S.* Typhi the *viaB* locus is positively regulated by the two-component regulator OmpR/EnvZ in response to osmotic tension. In elevated NaCl concentration the *viaB* locus is reported to be down-regulated. [11], [12], [13]. We quantified immuno-gold labelling with anti-Vi antibody following culture at 0.09 M and 0.3 M NaCl. Culture of *S.* Typhimurium C5.507 Vi^+^ in media containing 0.09 M NaCl resulted in ∼2-fold increase in labelling than that observed following culture in 0.3 M NaCl. Furthermore, a derivative in which the *ompR* gene was inactivated by deletion was not labelled, even when cultured in low osmolarity medium (LB+0.09 M NaCl). The quantification of labelling with anti-Vi^+^ coated gold beads correlated with the lack of agglutination with anti-Vi serum. Together these data indicate that the entire SPI-7 region of *S.* Typhi is integrated into the *S.* Typhimurium genome in C5.507 Vi^+^ and the pattern of expression of Vi antigen is similar to that in *S.* Typhi.

### Expression of Vi polysaccharide by *S.* Typhimurium does not impact colonisation of C57BL/6 mice following oral inoculation

**Figure 4:**
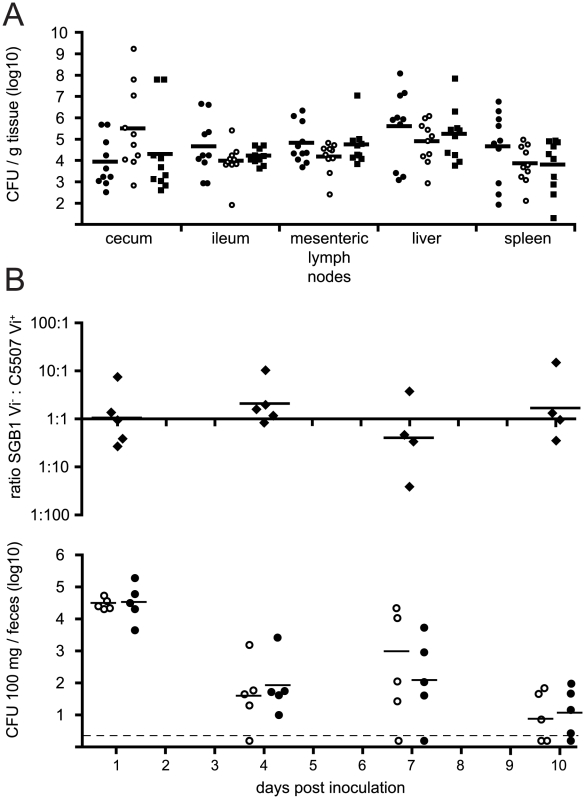
Colonisation of C57BL/6 mice with *S.* Typhimurium C5, *S.* Typhimurium C5507, and *S.* Typhimurium SGB1 (C5507 D*tviB*::kan^r^). (A) Groups of ten C57BL/6 genetically susceptible mice were inoculated orally with 1×10^8^ cfu of *S.* Typhimurium C5 (closed circles), *S.* Typhimurium C5507 (open circles), or *S.* Typhimurium SGB1 (C5507 D*tviB*::kan^r^) (closed squares). Horizontal bar indicates the geometric mean. Mice were culled on day 5 post-inoculation and the cfu in mesenteric lymph nodes, cecum, ileum, spleen and liver homogenates determined. (B) A group of five 129/sv genetically resistant mice were inoculated orally with an equal mixture of 1×10^9^ CFU *S.* Typhimurium C5507 Vi^+^, and *S.* Typhimurium SGB1 Vi^−^ (C5507 D*tviB*::kan^r^). The mean log10 ratio of these two strains in fresh fecal pellets on days 1, 4, 7 and 10 post inoculation are plotted (top), the CFU per 100 mg of *S.* Typhimurium C5507 Vi^+^ (open circles) and *S.* Typhimurium SGB1 Vi^−^ (filled circles) are plotted (below).

*S.* Typhi are host-adapted to higher primates and attenuated in mice following inoculation by the oral or parenteral routes. Consequently, we determined if *S.* Typhimurium C5.507 Vi^+^ could colonise the genetically susceptible C57BL/6 mouse. Mice were inoculated by oral gavage with approximately 1×10^8^ CFU *S.* Typhimurium C5, C5.507 Vi^+^ or SGB1 (Δ*tviB*). No significant difference in the colonisation of MLN, ceacum, ileum, spleen or liver was observed for these derivatives five days post inoculation (Figure 4A). To further determine the effects of the Vi capsule on chronic colonization as well as shedding within the faeces, we inoculated 129/sv mice by oral gavage with a mixture containing approximately 1×10^9^ CFU C5.507 Vi^+^ or SGB1. C5.507 Vi^+^ and SGB1 Vi^−^ were shed in the stool at similar levels on day 1, 4, 7 and 10 post inoculation (Figure 4B).

### Infection with *S.* Typhimurium C5.507 Vi^+^ results in altered innate immune cell population in spleen compared to a Vi-negative derivative

**Figure 5:**
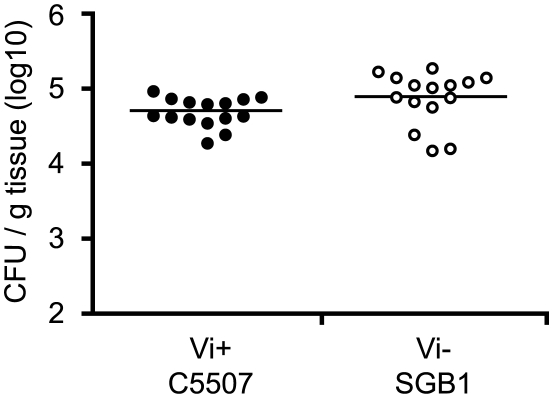
Colonisation of C57BL/6 mouse spleen by *S.* Typhimurium C5507, and *S.* Typhimurium SGB1 (C5507 D*tviB*::kan^r^). Groups of fifteen C57BL/6 mice were inoculated orally with 1×10^5^ cfu of *S.* Typhimurium C5507 (closed circles) or *S.* Typhimurium SGB1 (C5507 D*tviB*::kan^r^) (open circles). Mice were culled on 24 hours post-inoculation and the cfu in spleen homogenates determined.

To determine the impact of Vi expression on early innate immune responses to *S.* Typhimurium infection, spleens and MLN from naïve mice or from mice given a single i.v. or oral dose of C5.507 Vi^+^ or SGB1 *tviB* (Vi^−^) were examined by flow cytometry 24 hours post-inoculation. This time point was chosen since we were interested in determining the early innate immune response and because Vi is expressed on the surface of C5.507 Vi^+^ during this period but is down regulated by four days post inoculation [14]. Interestingly, mice inoculated with the SGB1 Vi^−^, had a small but significant increase in the levels of bacterial spleen colonisation (p = 0.025, unpaired, two tail, Mann Whitney) at 24 h compared to mice infected with C5.507 Vi^+^ (Figure 5).

**Figure 6:**
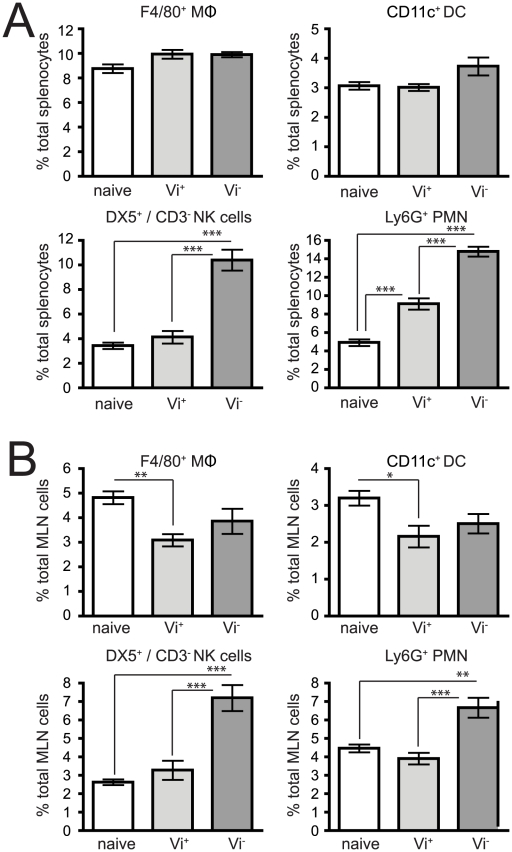
Expression of the Vi capsule induces differential innate immune responses shortly after infection. Cells were isolated from the spleens after i.v. infection (A) or MLN after oral infection (B) of C57BL/6 mice 24 h after infection with C5507 (Vi^+^) or SGB1 (Vi^−^) *S.* Typhimurium and stained with flurochrome-labelled mAb and analysed by flow cytometry in which 20,000–200,000 events were recorded. Columns represent the percentage ± SEM. Significant differences in values of * p<0.05; **, p<0.01; ***, p<0.001, as determined by one-way ANOVA followed by Bonferroni's multiple comparison test.

Examination of the spleen population of CD11c^+^ (dendritic cells, DC), F4/80^+^ (macrophage, MΦ), DX5^+^/CD3^−^ (natural killer, NK) cells and Ly6G^+^ (polymorphonuclear, PMN) in C5.507 Vi^+^ or SGB1 (Vi^−^) infected mice revealed differences in immune cell populations during early infection that correlated with the presence of a functional Vi locus (Figure 6A and Table 1). Infection with C5.507 Vi^+^ resulted in moderate but significant increases (p<0.05) in both percentage and total cell numbers of PMN in spleens at 24 h when compared to naïve animals, although other immune cells monitored were largely unchanged. In contrast, spleens from mice at 24 h after inoculation with SGB1 Vi^−^ had dramatically increased (p<0.001) percentage of PMN and NK cells, a significant increase (p<0.001) in the total number of DC and MΦ, although as a percentage they were not different form the population of these cells in spleen from naïve animals. We also observed an increase in the percentage of NK and PMN cells in mice infected with SGB1 Vi^−^ compared to C5.507 Vi^+^ and an increase in the total number of both DC and MΦ populations.

**Table 1:** Innate cell numbers after infection with Vi^+^ or Vi^−^ *S.* Typhimurium.

tissue	cell type	naïve	Vi+	Vi^−^
spleen	F4/80^+^ MΦ	393.7±43.6	587.1±65.2	878.5±89.1*** †
	CD11c^+^ DC	138.4±15.3	176.8±19.1	314.8±21.2*** †††
	DX5^+^/CD3^−^ NK cells	151.4±18.6	238.2±40.1	891.7±82.3*** †††
	Ly6G^+^ PMN	223.4±29.2	545.7±75.7*	1299±115.5*** †††
MLN	F4/80^+^ MΦ	9.6±1.3	13.1±1.4	22.2±2.8*** ††
	CD11c^+^ DC	6.1±0.6	9.3±1.7	15.2±2.1*** †
	DX5^+^/CD3^−^ NK cells	5.2±0.7	14.3±3.0	41.5±3.4*** †††
	Ly6G^+^ PMN	8.7±1.0	16.8±2.3	40.2±8.9*** †††

Total cell number for spleen (i.v. infection) or MLN (oral infection) after 24 h (1×10^4^) ± SEM.

*indicates significant values of p<0.05;

**, p<0.01;

***, p<0.001, as determined by one-way ANOVA followed by Bonferroni's multiple comparison test when compared to naïve mice.

**†:** indicates significant values of p<0.05;

**††:** , p<0.01;

**†††:** , p<0.001, as determined by one-way ANOVA followed by Bonferroni's multiple comparison test when Vi^+^ are compared to Vi^−^ infected mice.

During natural infection following oral ingestion *S.* Typhimurium invades the enterocytes and M cells of the terminal ileum and then enters the lymph system that drains *via* the mesenteric lymph nodes (MLN). We therefore determined the impact of Vi expression on the innate immune cell populations of the MLN 24 hours after oral infection (Figure 6B and Table 1). Similar but not identical patterns of splenocyte immune cell populations were observed. For example, mice inoculated with SGB1 Vi^−^ had a dramatically increased (p<0.001) percentages of PMN and NK cells in the MLN compared with MLN from C5.507 Vi^+^ infected or naïve mice. However, in contrast to splenocyte population in i.v. inoculated mice in which we observed a significant increase in the percentage of PMN with C5.507 Vi^+^, PMN in MLN following infection with C5.507 was not significantly different from naïve animals.

### Vi expression modulates cytokine and chemokine responses

**Figure 7:**
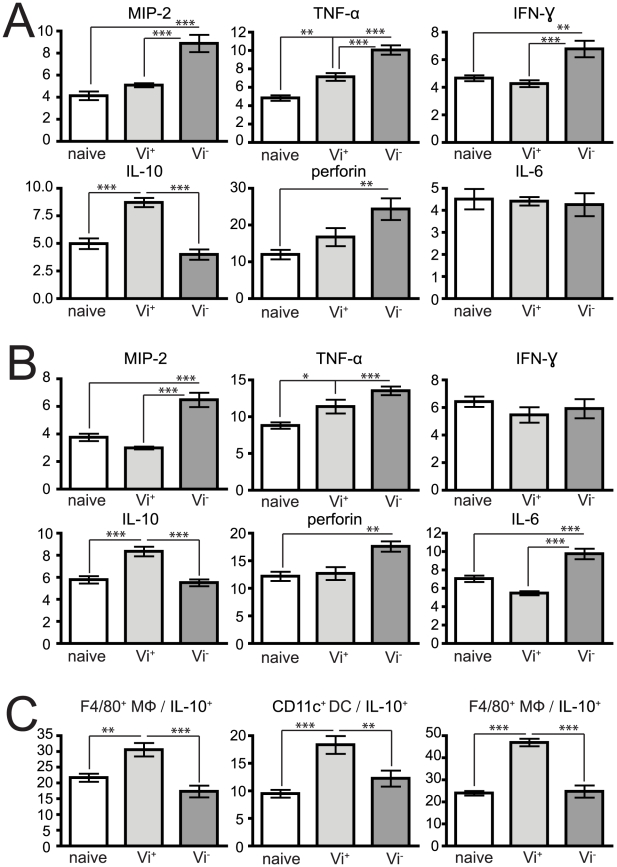
Vi expression also impacts on the cytokine profile of cells after *S.* Typhimurium infection. Isolated splenocytes after i.v. infection (A) or MLN after oral infection (B) from naïve, or 24 h infected C5507 (Vi^+^) and SGB1 (Vi^−^) mice were stimulated for 6 h with BD Leukocyte Activation Cocktail with BD GolgiPlug (BD Biosciences) *in vitro*, permeabilised and stained with anti-cytokine flurochrome-labelled mAb. (C) Cells were stained with mAb to specific surface markers F4/80^+^, CD11c^+^ and DX5^+^/CD3^−^ the identity of innate populations, permeabilised and stained with anti-IL-10. Data represent percent of cytokine positive cells out of total spleen populations ± SEM. Significant differences in values of * p<0.05; **, p<0.01; ***, p<0.001, as determined by one-way ANOVA followed by Bonferroni's multiple comparison test.

The intracellular cytokine response to Vi^+^ and Vi^−^ *S.* Typhimurium were determined following *ex vivo* stimulation of splenocyte and MLN cells with phorbol 12-myristate 13-acetate (PMA). Flow cytometric analysis of intracellular cytokine expression in both splenocytes (after i.v. infection) and MLN cells (after oral infection) from *Salmonella*-infected mice were determined (Figure 7 and Table 2). Infection with SGB1 Vi^−^ was associated with a significant increase (p<0.05) in the percentage and total cell number of MIP-2, TNF-α, IFN-γ and perforin producing splenocytes cells when compared to similarly stimulated naïve cells. In the case of MIP-2, IFN-γ and perforin producing cells from mice infected with C5.507 Vi^+^ the levels were not significantly different than those from naïve animals. Additionally, although, we did observe a significant increase in the proportion of TNF-α positive cells compared to naïve animals this was significantly lower (p<0.05) than observed in cells from mice infected with SGB1 Vi^−^. There was no observed difference in IL-6 expression by splenocytes from naïve mice or mice infected with C5507 Vi^+^ or SGB1 Vi^−^.

**Table 2:** Total number of cytokine producing cells after infection with Vi^+^ or Vi^−^ *S.* Typhimurium.

tissue	cytokine	naïve	Vi^+^	Vi^−^
spleen	MIP-2	103.4±12.7	190.5±19.5	562.2±80.3*** †††
	TNF-α	129.1±18.1	272.6±36.5	625.4±57.3*** †††
	IFN-γ	122.1±17.9	163.3±22.7	428.5±57.1*** †††
	IL-10	129.9±20.6	323.1±30.9***	246.6±30.2*
	perforin	311.3±47.4	637.2±113.3	1529.0±240.5*** ††
	IL-6	114.4±15.0	165.6±16.1	269.5±46.7**
MLN	MIP-2	7.5±0.6	11.6±1.1	35.8±4.2*** †††
	TNF-α	18.5±2.4	44.7±5.5**	74.1±5.5*** †††
	IFN-γ	13.7±1.9	21.2±3.0	31.2±2.9*** †
	IL-10	11.9±1.3	32.2±2.9***	30.5±2.7***
	perforin	24.3±3.2	51.8±5.4	106.3±12.8*** †††
	IL-6	14.2±1.4	20.9±1.4	54.1±5.4*** †††

Total cell number for spleen (i.v. infection) or MLN (oral infection) after 24 h (1×10^4^) ± SEM.

*indicates significant values of p<0.05;

**, p<0.01;

***, p<0.001, as determined by one-way ANOVA followed by Bonferroni's multiple comparison test when compared to naïve mice.

**†:** indicates significant values of p<0.05;

**††:** , p<0.01;

**†††:** , p<0.001, as determined by one-way ANOVA followed by Bonferroni's multiple comparison test when Vi^+^ are compared to Vi^−^ infected mice.

Since the natural route of infection for *Salmonella* is via the oral route we also determined the impact of infection with *S.* Typhimurium C5507 Vi^+^ and SGB1 Vi^−^ on intracellular production of MIP-2, TNF-α, IFN-γ and perforin by immune cells of the MLNs 24 h post inoculation. Similar observations were made to those found in splenocytes following i.v. inoculation but differences in IFN-γ^+^ cell populations and IL-6 producing cells were observed. IFN-γ^+^ splenocytes were elevated, but not IFN-γ^+^ MLN cells, and significantly greater percentages and numbers of IL-6 producing MLN cells, but no significant differences in the IL-6 splenocyte population in SGB1 Vi^−^ infected mice when compared to naïve animals. With respect to SGB1 Vi^−^ infected mice, we also detected a significant increase (p<0.05) in the percentage of TNF-α producing splenocytes and MLN cells.

We also determined the cellular source of these cytokines within the lymphoid tissues after infection (Table 3). Splenocytes isolated from SGB1 Vi^−^ infected mice were found to have significantly more MIP-2^+^ and IFN-γ^+^ (p<0.001) producing cells compared to both naive and C5.507 Vi^+^ infected mice. TNF-α was mainly expressed by macrophage and NK cells and to a lesser extent PMN. IL-6 expression was only significantly different in NK cells in SGB1 Vi^−^ infected compared toC5.507 Vi^+^ infected or uninfected mice. Also the numbers of macrophage detectably producing IL-6 from Vi^+^ infected mice was significantly (p<0.05) lower than both naïve and Vi^−^ infected mice. The numbers of perforin^+^ NK cells was significant higher (p<0.001) in those mice infected with SGB1 Vi^−^ compared to naïve and C5.507 Vi^+^ infected mice. A similar cytokine profile was also observed within the MLN from orally infected mice. Notably, the cellular sources of the significant increase in IL-6^+^ MLN cells from SGB1 Vi^−^ infected mice included DC, MΦ and NK cells (data not shown).

**Table 3:** Vi impacts on the cytokine profile of innate cells shortly after infection.

cell type	cytokine	naïve	Vi^+^	Vi^−^
F4/80^+^ MΦ	MIP-2	21.2±1.7	18.4±1.5	35.7±1.4*** †††
	TNF-α	25.7±0.8	29.6±0.6	37.8±1.3*** ††
	IFN-γ	10.2±0.5	11.9±0.5	17.2±1.1*** †††
	IL-6	24.0±1.0	18.4±1.5*	23.7±1.0†
CD11c^+^ DC	MIP-2	8.1±0.7	7.9± 0.8	14.7±1.0*** †††
	TNF-α	12.8±1.4	12.2±0.7	15.5±1.2
	IFN-γ	15.2±0.4	16.3±0.8	20.6±0.8*** †††
	IL-6	13.1±0.7	15.4±1.2	13.4±1.0
DX5^+^/CD3^−^ NK cells	MIP-2	14.4±1.1	8.7±0.5***	18.3±0.9** †††
	TNF-α	14.5±1.2	20.9±1.6*	30.0±1.7*** †††
	IFN-γ	18.9±1.2	18.4±1.8	27.5±1.0*** †††
	IL-6	8.9±1.0	10.2±0.9	16.8±1.5*** †
	perforin	38.9±1.6	48.5±2.1	66.8±3.7*** †††
Ly6G^+^ PMN	MIP-2	8.0±0.4	6.6±0.4	11.1±1.1* †††
	TNF-α	23.4±0.9	25.6±1.3	29.2±0.9*
	IFN-γ	15.2±0.8	14.6±0.7	22.1±1.0* †
	IL-6	19.8±1.7	18.2±1.4	20.9±1.1

Cells were stained with mAb to specific surface markers F4/80^+^, CD11c^+^, DX5^+^/CD3^−^ and Ly6G^+^ to identify innate populations, permeabilised and stained with anti-MIP-2, TNF-α, IFN-γ, perforin and IL-6. Data represent percent of cytokine positive cells out of total spleen populations ± SEM.

*indicates significant values of p<0.05;

**, p<0.01;

***, p<0.001, as determined by one-way ANOVA followed by Bonferroni's multiple comparison test when compared to naïve mice.

**†:** indicates significant values of p<0.05;

**††:** , p<0.01;

**†††:** , p<0.001, as determined by one-way ANOVA followed by Bonferroni's multiple comparison test when Vi^+^ are compared to Vi^−^ infected mice.

Strikingly, infection with C5507 Vi^+^ was also associated with a significant increase in the percentage and total number of cells producing the anti-inflammatory cytokine IL-10 when compared to both stimulated naïve and SGB1 Vi^−^ infected cells. Indeed, no increase in the number of cells producing IL-10 above that in naïve resulted from infection with SGB1 Vi^−^. We observed that re-stimulated MΦ, DC and NK cells, but not PMN, from C5.507 Vi^+^ infected mice expressed significantly more (p<0.01) IL-10 when compared to similarly stimulated naïve and Vi^−^ infected splenocytes (Figure 7C).

### Vi expression modulates *in vivo* innate immune responses in a TLR-dependent manner

**Figure 8:**
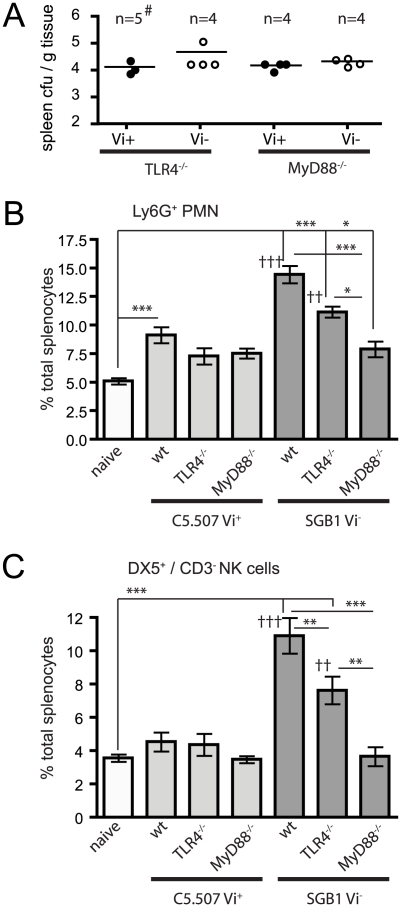
Vi modulates innate immune responses through TLR. Spleens were removed from naïve, WT or KO animals (both MyD88^−/−^ and TLR4^−/−^) infected with C5.507 (Vi^+^) or SGB1 (Vi^−^) 24 h after infection. The cfu per g of tissue (A) and the percentage of splenocytes that were Ly6G^+^ (B) or DX5^+^/CD3^−^ (C) were determined. Columns represent the percentage of cells ± SEM. Significant differences are indicated * p<0.05; **, p<0.01; ***, p<0.001, and for comparison of Vi^+^ compared to Vi^−^ infected mice, ^††^ p<0.01; ^†††^, p<0.001, as determined by one-way ANOVA followed by Bonferroni's multiple comparison test. # Colonisation data for two of the mice in the TLR4^−/−^ mouse group inoculated with C5.507 were not determined.

To determine if the observed differences in the innate immune response in mice infected with Vi^+^ *S.* Typhimurium were due to detection of PAMPs by TLRs, we infected both MyD88^−/−^ and TLR4^−/−^ mice with SGB1 Vi^−^ or C5507Vi^+^. Both bacterial colonisation and splenocyte PMN and NK cell populations were examined 24 h post-infection in wild type and KO mice (Figure 8 and Table 4).

**Table 4:** Total number of PMN and NK cells after infection with Vi^+^ or Vi^−^ *S.* Typhimurium in TLR4^−/−^ and MyD88^−/−^ mice.

cell type	naïve	WT Vi^+^	TLR4^−/−^ Vi^+^	MyD88^−/−^ Vi^+^	WT Vi^−^	TLR4^−/−^ Vi^−^	MyD88^−/−^ Vi^−^
Ly6G^+^ PMN	196.6±21.2	597.3±78.7*	399.2±73.3	454.1±47.0	1580.0±130.9**	909.6±105.0** ††	501.9±64.1††
DX5^+^/CD3^−^ NK	133.2±17.2	261.4±39.4	193.3±13.7** ††	267.0±41.7	1149.1±143.9**	909.6±105.0** ††	241.9±64.4††

Total cell number of PMN and NK cells in the spleen 24 h post (×10^4^) ± SEM.

*indicates significant values of *, p<0.01;

**, p<0.001, as determined by one-way ANOVA followed by Bonferroni's multiple comparison test when compared to naïve mice.

**†:** indicates significant values of †, p<0.01;

**††:** , p<0.001, as determined by one-way ANOVA followed by Bonferroni's multiple comparison test when WT Vi^−^ are compared to KO Vi^−^ infected mice.

As observed previously, spleens from wild type mice infected with C5.507 Vi^+^ had significantly increased percentage and number of PMN compared to naïve mice (p<0.01). In contrast, in both TLR4^−/−^ and MyD88^−/−^ mice infected with C5.507 Vi^+^ the numbers of PMN was not significantly different (p>0.05) in percentage or total cell number compared with naïve mice. Indeed, overall, in TLR4^−/−^ and MyD88^−/−^ infected mice the PMN and NK cell response to C5.507 Vi^+^ infection was indistinguishable from naïve mice. In contrast, although the response to SGB1 Vi^−^ infection in TLR4^−/−^ and MyD88^−/−^ was reduced compared to that in WT mice, there was still a significant response compared to naïve mice.

Infection of wild type mice with SGB1 Vi^−^ as before resulted in a significant increase (p<0.001) in the PMN population compared to both naïve and C5.507 Vi^+^ infected mice. Furthermore, unlike infections with C5.507 Vi^+^, both TLR4^−/−^ and MyD88^−/−^ mice infected with SGB1 Vi^−^ also had significantly more (p<0.05) PMN within their spleen compared with naïve mice. Indeed, when wild type TLR4^−/−^ and MyD88^−/−^ mice infected with SGB1 Vi^−^ were compared, both TLR4^−/−^ and MyD88^−/−^ mice had significantly less (p<0.001) PMN than wild type infected mice. Furthermore MyD88^−/−^ mice had a significant reduction (p<0.05) compared to TLR4^−/−^ SGB1 Vi^−^ infected mice. Importantly, there were also significantly fewer (p<0.01) PMN in C5.507 Vi^+^ infected TLR4^−/−^ mice compared to SGB1 Vi^−^ infected mice, but not within the MyD88^−/−^ groups.

The splenic NK cell population showed very similar responses to Vi^+^ and Vi^−^ *S.* Typhimurium PMN population in wild type and TLR4^−/−^ and MyD88^−/−^ mice (Figure 8B). Specifically, only Vi^−^ infected wild type and TLR4^−/−^ infected mice had significantly greater percentages and numbers of NK cells compared to naïve and C5.507 Vi^+^ infected mice. Additionally, both TLR4^−/−^ and MyD88^−/−^ SGB1 Vi^−^ infected mice had significantly lower levels of NK cells when compared to similarly infected wild type mice. MyD88^−/−^ infected mice also had significantly less NK cells when compared to TLR4^−/−^ SGB1 Vi^−^ infected mice. We again observed that C5.507 Vi^+^ infected TLR4^−/−^ mice had significantly less (p<0.01) NK cells than similar SGB1 Vi^−^ infected mice.

### Vi associated IL-10 expression regulates activation and chemotaxis of splenocytes *in vitro*

**Figure 9:**
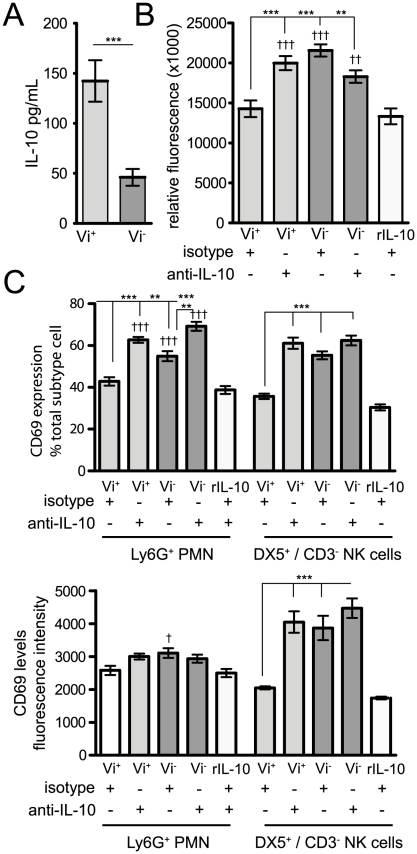
Vi induction of IL-10 impacts on chemotaxis and activation of splenocytes *in vitro*. (A) Spleen cells were isolated from naïve mice and stimulated for 24 h with 1∶1 ratio of Vi^+^ or Vi^−^ *Salmonella* strains and supernatants were analysed for levels of IL-10. Mean ± SEM are presented from six individual animals. Significance was determined using Mann-Whitney test, *** p<0.001. (B) Splenocytes were also stimulated with the various *S.* Typhimurium strains in the presence of anti-IL-10, isotype control antibody or rIL-10. Cells were then stained with surface antibodies and the percentage and expression/mean fluorescent intensity (MFI) of the activation marker CD69 on NK cells and PMN was analysed by flow cytometry. Columns represent the percentage of MFI ± SEM and significant differences were determined as in Figure 6. (C) Chemotactic migration of splenocytes towards supernatants from *Salmonella* stimulated cultures (in the presence of anti-IL-10, control antibody or rIL-10). After 4 h incubation, the level of cell migration was determined by reading the relative fluorescent units (RFU) at 480/520 nm. Data represents mean ± SEM minus negative (media alone) controls. Significant differences in values of * p<0.05; **, p<0.01; ***, p<0.001, as determined by Kruskal-Wallis followed by Dunn's multiple comparison test or ^†^ indicating significant values when compared to r-IL-10.

Splenocytes purified from naïve C57BL/6 mice were cultured with C5.507 Vi^+^ or SGB1 Vi^−^ bacteria for 24 h and the supernatant assayed for the presence of IL-10. The culture supernatant from cells stimulated with C5.507 Vi^+^ contained higher (p<0.001) IL-10 levels than SGB1 Vi^−^ stimulated splenocytes (Figure 9A). To address directly if enhanced expression of IL-10 associated with C5.507 Vi^+^ infection was at least in part responsible for the observed immune suppression in infected mice, splenocytes from naïve mice were stimulated with Vi^+^ or Vi^−^ *S.* Typhimurium in the presence of anti-IL-10 or isotype control antibody. Decreased chemotaxis (p<0.01) was observed in SGB1 Vi^−^ stimulated isotype treated cultures compared to both isotype control antibody and anti-IL-10 SGB1 Vi^−^ stimulated splenocytes. Notably, when C5.507 Vi^+^ stimulated cultures were grown in the presence of anti-IL-10 we no longer observed a significant reduction (p>0.05) in the movement of cells (Figure 9B). C5.507 Vi^+^ co-culture with isotype antibody significantly reduced (p<0.01) both the percentage and mean fluorescent intensity (MFI), expression levels, of the early activation marker CD69 on both PMN and NK cells. Again, presence of anti-IL-10 in C5.507 Vi^+^ stimulated cultures led to an increase in CD69 expression that was not significantly different (p>0.05) from SGB1 Vi^−^ stimulated splenocytes (Figure 9C). The addition of anti-IL-10 did not appear to have any significant effect on migration, although we did observe a significantly greater percentage of CD69^+^ PMN in anti-IL-10 SGB1 Vi^−^ treated cultures compared to isotype controls (Figure 9B and C). Further, addition of rIL-10 to naïve splenocytes gave a similar chemotaxis and immune activation profile (p>0.05) to that observed in C5.507 Vi^+^ stimulated splenocytes containing control antibody (Figure 9B and C).

## Discussion

In this study we addressed the hypothesis that early innate immune responses to *Salmonella* can be modulated at systemic sites by the expression of Vi. We exploit a *S.* Typhimurium/*S.* Typhi chimera (C5.507) harbouring ∼300 kb of the *S.* Typhi genome including the entire SPI-7 island containing the *viaB* locus. In our assays the colonisation level of mice following oral inoculation with this chimera strain was indistinguishable from the parent strain lacking the *S.* Typhi genomic region. However, we cannot discount small differences in colonisation that may be detectable in more sensitive experiments. Importantly the *viaB* locus in this strain is encoded as a single copy and in the natural genomic context. Consequently, the Vi polysaccharide capsule is expressed on the surface of C5.507 Vi^+^ at similar levels to that in *S.* Typhi and expression is controlled by osmotic stress in an OmpR dependent manner as in *S.* Typhi [11]. The pathogen chimera approach could be used to study other *S.* Typhi specific determinants or to overcome technical constraints associated with other host-adapted pathogens.

An immune-modulator role for Vi has been proposed previously based on observations of the effect of the expression of this polysaccharide on cytokine responses in T84, THP-1 and HEK293 cells in tissue culture [15], bone-marrow derived macrophage [10], and in an in vivo colitis models [9]. In these studies determination of relative transcription of IL-6, TNF-α, or IL-17 genes was generally used as a measure of the immune response to infection. Here we use an alternative approach to directly measure both the population of immune cells (macrophage, dendritic cells, NK cells and polymorphonuclear cells), present in the spleen of mice infected with *S.* Typhimurium C5.507 Vi^+^, and directly determine the intracellular level of a series of key inflammatory or anti-inflammatory cytokines; MIP-2, TNF-α, IFN-γ, IL-6 and IL-10. When we compared the splenocyte population 24 h after infection we observed significantly fewer PMN in mice inoculated with C5.507 Vi^+^ compared to SGB1 Vi^−^. This could be explained by the observation that a greater number of TNF-α and MIP-2 producing MΦ, NK cells and DC were found in those mice infected with the Vi^−^ compared to the Vi^+^ *S.* Typhimurium. Through neutrophil recruitment and activation, TNF-α and MIP-2 are known to be important mediators of intestinal inflammation and associated pathology, and the difference in the induction of these mediators by *S.* Typhi and *S.* Typhimurium may explains some of the differences in disease outcome associated with these pathogens [9]. We also report that Vi expression is associated with decreased TNF-α and MIP-2 expression in the spleen of infected mice following inoculation by the intravenous route. Vi mediated decrease in IL-6 production by bone marrow derived macrophages in vitro has been described previously [10]. However, we only observed reduced IL-6 expression in cells (MΦ, DC and NK cells) of the mesenteric lymph node following oral inoculation but no detectable difference in intracellular IL-6 levels in mice infected via the intravenous route regardless of Vi expression. Notably, our data shows that reductions in neutrophil chemo attractants directly impacts on their trafficking *in vivo*. NK cells were also influenced by the expression of Vi following either intravenous or oral infection with C5.507 Vi^+^ as NK cell populations were not significantly different from those in naïve mice. In contrast, mice infected with SGB1 Vi^−^ showed a marked increase in the NK cell population. We also observed an increase in the proportion of perforin positive NK cells. Perforin is stored in NK and CD8^+^ T cells as granules and is key to their ability to destroy infected host cells. The reduction in NK cell influx and associated decrease in perforin in response to C5.507 Vi^+^ infection may also explain in part how expression of this polysaccharide contributes to the ability of the *S.* Typhi to disseminate to systemic sites, colonise and replicate.

The effect of Vi expression by *S.* Typhimurium on DC and MΦ populations was not as great as that observed for PMN and NK cell populations. Nonetheless, we did observe significantly lower numbers of these innate cells in mice infected with Vi^+^ compared to Vi^−^ *S.* Typhimurium. The apparent impact of Vi expression on the infux of DC and MΦ may also be attributed to MIP-2 since this chemokine can also modulate the trafficking of DC [16]. As well as being a chemo attractant in its own right, TNF-α induces the synthesis of a number of chemokines, including IP-10, RANTES, KC, in a cell-type and tissue-specific manner [17]. Impairment of this response may also account for the reduction in splenic MΦ and NK cells observed in C5.507 Vi^+^ infected mice. Together, these data support a role for Vi in modulating the recruitment of immune cells to sites beyond the intestinal mucosa, including the mesenteric lymph nodes and spleen.

The mechanism by which Vi modulates innate responses is currently not known, although it has been postulated that Vi may mask LPS therefore preventing its detection by TLR4 [10]. We report that spleen from TLR4^−/−^ and MyD88^−/−^ mice infected with SGB1 Vi^−^ have reduced immune cell splenocyte populations compared to wild type control mice. However, there were significantly greater immune cell populations in TLR4^−/−^ and MyD88^−/−^ mice than in naïve mice suggesting that proinflammatory signals other than those dependent on these were in operation. These data suggest Vi may play a role as a physical barrier separating *Salmonella* PAMPs from TLR4 activation. However, data from MyD88^−/−^ mice suggests that while TLR4 signalling plays some role in the immuno-modulatory aspect of Vi, other pathways may also be involved.

Much of what is known of the immune-modulator effects of Vi is from observations of induction of pro-inflammatory cytokines and chemokines. We additionally examined the production of an anti-inflammatory cytokine, IL-10, and report a significant increase in the number of IL-10-expressing cells in mice infected with Vi^+^ compared to Vi^−^ *S.* Typhimurium. In part, IL-10 acts on macrophage and myeloid dendritic cells to inhibit the development of a T_H_-1 response (reviewed in [18]). IL-10 is produced by many different cell types of the innate and adaptive immune systems, and the temporal and spatial expression is likely important to moderating immune response [19]. Importantly, IL-10 is induced in macrophage and myeloid dendritic cells by a variety of pathogen derived products, including LPS following detection by TLR-4 [20]. We did not observe induction of IL-10 in splenocytes from mice infected with SGB1 Vi^−^ relative to naïve mice at the time point studied, but induction was specifically observed in mice infected with C5.507 expressing Vi. Indeed, when we determined the innate populations producing IL-10 we observed that MΦ, DC and NK cells were the cell types responsible for this increased IL-10 phenotype. Importantly, we also observed that inhibition of IL-10 from C5.507 Vi^+^ stimulated splenocytes *in vitro* directly impacted both chemotaxis and activation status of immune cells. Notably, previous studies have shown that IL-10 can inhibit chemokine expression and reduce iNOS production from PMN [21], [22]. Therefore, early production of IL-10 after infection with Vi^+^ *Salmonella* may be important in diminishing neutrophil influx and activation and thereby increasing the ability of this pathogen to efficiently colonise and infect distal sites within the host. At later infection time-points Vi may be an important mechanism associated with dampening the T_H_-1 response that is normally associated with resolving infections by invasive bacterial pathogens. Indeed, higher levels of IL-10, as well as IL-4, have been detected in PBMC culture of typhoid fever patients when compared to healthy control subjects.

*S.* Typhimurium C5507 Vi^+^ provides a model pathogen to study the impact of Vi and potentially other *S.* Typhi specific virulence genes encoded on SPI-7, in the well characterized murine typhoid model. Recently, two excellent murine models for typhoid fever have been described [23], [24]. The utility of *S.* Typhimurium C5507 Vi^+^ as a model system has previously been demonstrated in studies to determine the protection afforded by a conjugated Vi subunit vaccine [25]. Furthermore, *S.* Typhimurium C5507 Vi^+^ has been used to studies expression of Vi polysaccharide capsule from heterologous promoters including P*ssaG*, to improve the anti-Vi immune response to live oral vaccines against typhoid (unpublished observations). However, observations from the use of a surrogate infection models should be considered with care. Many differences between the chimera infection of mice remain compared with the natural *S.* Typhi infections of humans. One of these is that rate at which the infection proceeds and the dynamics of colonisation and clearance. These differences are likely accounted for in the genetic differences outside of the chimera region and also differences in the host species.

Together these data describe the impact of Vi expression on the outcome and innate immune response to *S.* Typhimurium infections in mice. Modulation of the innate immune response by the Vi polysaccharide capsule decreased innate cell recruitment and provided insights into how *S.* Typhi effects its interaction with the host to provide the desired outcome from the host-pathogen interaction. This is consistent with a pathogenic strategy central to which is a slowly progressing systemic disease that is rarely associated with death from sepsis or cytokine storm, but that ultimately provides access for this pathogen to the gall bladder and potentially other immune privileged niches, required for chronic carriage.

## Materials and Methods

### Bacterial strains and culture conditions

*S.* Typhimurium C5507 Vi^+^ was a gift from M. Popoff (Institute Pasteur, France) and was generated by conjugation of *S. enterica* Typhi Ty2 with *S. enterica* Typhimurium C5. A serotype Typhimurium strain in which the *phoN* gene was replaced by the *aph* gene was constructed by transferring the *aph* gene from AJB715 into strain SL1344 by P22 transduction. This derivative was designated AMJ204. SGB1 Vi− strain was generated by precise deletion of the tviB gene and replacement of this with the aph gene that confers resistance to kanamycin using the Datsenko and Wanner red recombinase allelic exchange methodology [26]. Oligonucleotide primers 5′ GCCAGAACCAGTTTGGTCCGTAGTTCTTCGTAAGCCGTCATGATTGTGTAGGCTGGAGCTGCTTCG 3′ and 5′ AATTAACTTTGTAAATATAAAATTTTAGTAAAGGATTAATAAGAGCATATGAATATCCTCCTTAG 3′ were used to amplify the *aph* gene from pKD4 template. Strain SGB1 expressed comparable amounts of flagellin on culure in LB broth in vitro as determined by crude flagella preparation and separation of flagellin monomer by SDS PAGE (data not shown). Bacteria were cultured aerobically at 37°C in Luria-Bertani (LB) broth or LB with 15% agar supplemented with antibiotics at appropriate concentrations; ampicillin (Amp), 100 mg/L (LB+Amp); chloramphenicol (Chl), 30 mg/L (LB+Chl); and kanamycin (Kan), 30 mg/L (LB+Kan).

### Sequence analysis

To identify the boundaries of *S.* Typhi and *S.* Typhimurium-derived genomic sequence, genomic DNA was prepared from *S.* Typhimurium C5507 and sequenced using the Illumina/Solexa Genome Analyser. Over 6.5 million single end reads of 36 bp were generated, giving a theoretical 48-fold coverage of the *S.* Typhimurium genome. Reads were mapped to the reference genomes *S.* Typhimurium LT2 (EMBL:AE006468) and *S.* Typhi CT18 (EMBL:AL513382) using Maq (maq.sourceforge.net), single nucleotide polymorphisms between C5507 and the reference genomes were identified. The position and density of these substitutions compared to the two reference genomes was visualised using Artemis and ACT (Artemis Comparison Tool) [27].

### Transmission Electron Microscopy (TEM)

Bacteria cultured for 24 hr at 37°c were sampled selecting a single colony from each strain, mixing with 20 µl sterile distilled water and rapidly freezing in a Bal-Tec HPM010 high pressure freezer. Samples for immunogold-labelling were freeze-substituted in a Leica EM AFS at −90°C in methanol containing 0.2% uranyl actetate and 0.1% glutaraldehyde followed by low temperature embedding in Lowicryl HM20 resin. Samples for ultrastructural analysis were freeze-substituted with acetone containing 0.1% tannic acid, 0.5% glutaraldehyde sequentially with acetone containing 1% osmium tetroxide and 0.1% uranyl actetate followed by room temperature embedding in TAAB 812 resin. 50 µm ultrathin sections were cut on a Leica EM UC6 and contrasted with lead citrate and uranyl acetate For immunogold-localisation ultrathin sections were labelled with anti Vi antibody and probed with protein A gold as previously described [28]. Images were taken on an FEI Tecnai Spirit 120 kV TEM with a Tietz F415 CCD camera. For quantification of labelling fifteen random clearly defined bacteria were selected and the number of associated 10 nm gold particles counted.

### Experimental infections of mice

Female wild-type (WT) C57BL/6 and 129/sv mice (6–8 week old) and knock-out (KO) C57BL/6 mice, including MyD88^−/−^ [29] and TLR4^−/−^ [30] were kind gifts and were bred at The Sanger Institute Research Support Facility (RSF). All animals were given food and water *ad libitum*. Mice were sacrificed by cervical dislocation or exsanguination. The *S.* Typhimurium strains were grown with shaking in LB broth (with appropriate antibiotics) for 20 h. Bacteria were then harvested by centrifugation, washed, and suspended in PBS (pH 7.4) to approximately 10^9^ CFU per ml. Groups of 5–10 mice were inoculated orally by gavage or intravenously with 0.2 ml of bacterial suspension. Viable counts were determined in the inoculum by serial dilutions onto LB agar. After 24 h or five days the mice were culled and their MLN, terminal end ileum, cecum, spleen, and liver were aseptically removed and weighed. Organs were homogenised in sterile water (5 ml) using a Seward Stomacher 80 (Seward, London UK) for 2 minutes at high speed. Serial dilutions of each organ were plated onto LB agar. Colonies were enumerated after overnight incubation at 37°C.

### Ethics statement

All animal procedures were performed in accordance with the United Kingdom Home Office Inspectorate under the Animals (Scientific Procedures) Act 1986. Ethical approval for these procedures were granted by the Wellcome Trust Sanger Institutes Ethical Review Committee.

### Flow cytometry and ELISA

Single cell suspensions from the spleens and MLN of individual mice were prepared to obtain a final concentration of 5×10^5^ cells/well in blocking buffer (1× PBS/1% BSA/0.05% sodium azide/1% rat, hamster and mouse serum). 0.05 ml of each mAb dye mix, 0.005 ml of the amine-reactive viability dye ViViD (Invitrogen) to determine dead cells, with incubation in the dark at 4°C for 30 minutes. The mAb used for flow cytometry were (BD Biosciences unless stated otherwise); CD11c, clone HL3 with PE-Cy7 conjugate (BD Biosciences), Ly6G, clone RB6-8C5 with PE conjugate (BD Biosciences), CD49b, clone DX5 with FITC or PE conjugate (BD Biosciences), F4/80, clone BM8 with TRI-COLOR conjugate (Invitrogen), CD3, clone 145-2C11 with APC, CD69, clone H1.2F3 with PE-Cy7, Alexa Fluor 700, conjugate (BD Biosciences), CXCL2/MIP-2, clone 40605 with biotin conjugate (AbD Serotec), IL-10, clone JES5-16E3 with APC conjugate (BD Biosciences), TNF-α, clone MP6-XT22 with PE-Cy7 conjugate (BD Biosciences), IL-6, clone MP5-20F3 with PE conjugate (BD Biosciences), IFN-γ, clone XMG1.2 with PE-Cy7 conjugate (BD Biosciences), perforin, clone eBioOMAK-D with FITC conjugate (eBioscience), streptavidin, with PE-Texas Red conjugate (BD Biosciences). Cells were washed twice with blocking buffer and finally resuspended in 0.2 ml 1% paraformaldehyde. To perform flow cytometric analyses and measure relative fluorescence intensities a FACSAria cytometer and BD Diva software (Becton Dickinson) were used. For each mouse 20,000–200,000 events were recorded. The percentage of cells labelled with each mAb was calculated in comparison with cells stained with isotype control antibody. Background staining was controlled by labelled isotype controls and fluorescence-minus-one (FMO). The results represent the percentage of positively stained cells in the total cell population exceeding the background staining signal. For determination of intracellular cytokine production by splenocytes, cells were incubated for 6 h at 37°C with BD Activation Cocktail (BD Biosciences) containing Phorbol 12-Myristate 13-Acetate (PMA), plus GolgiPlug or GolgiPlug alone (BD Biosciences). Cells were then washed with staining buffer and stained at 4°C with surface mAbs. Cells were then fixed and saponin-permeabilised (Perm/Fix solution, BD Biosciences) and incubated with cytokine mAb as listed above or isotypic controls. After 30 min cells were twice washed in permealisation buffer (BD Biosciences) and then analysed by flow cytometry as described above. For determination of IL-10 levels from in vitro stimulated splenocyte supernatants the IL-10 Instant ELISA (Bender MedSystems) system was used according to manufacturer's instructions.

### *In vitro Salmonella* stimulations

Spleen suspensions were prepared as described above and 2×10^5^ cells added to a 96-well round bottom plate and were left for 1 h at 37°C 5% CO_2_. Cells were then stimulated with appropriate 1∶1 ratio of *Salmonella* strains supplemented with 20 µg/mL anti-IL-10 or appropriate isotype control (PeproTech). In others, cells were supplemented with 100 ng/mL recombinant (r)-IL-10 (PeproTech). Splenocytes were also stimulated with LPS (100 ng/mL) or RPMI^+^ medium as positive and negative controls respectively. Cells were then incubated for approximately 24 h before being centrifuged at 800× g for 5 min. Some supernatant was removed and stored at −80°C for subsequent cytokine analysis. Remaining cells from stimulation were then stained for flow cytometry with surface (DX5^+^/CD3^−^ or Ly6G^+^) and the activation marker CD69 as described above.

### Chemotaxis assay

Migration of *Salmonella* stimulated splenocytes was analysed in 96-well QCM Chemotaxis Cell Migration Assay (Millipore, UK) with 5-µm pore polycarbonate filters. Briefly, 2×10^5^ cells in 0.1 ml of RPMI^+^ were placed into the migration chambers. Supernatant from stimulated splenocytes were added to the lower chamber. Migration was performed for 4 h at 37°C with 5% CO_2_. Cells/media from the top chamber of the insert was discarded and remaining cells removed using Cell Detachment Solution (Millipore, UK). Lysis Buffer/Dye Solution (Millipore, UK) were added to each well and incubated for 15 min. 0.15 ml of this mixture was then transferred to new 96-well plate and read with a fluorescence plate reader using 480/520 nm filter set.

### Statistical analysis

Experimental results were plotted and analysed for statistical significance with Prism4 software (GraphPad, San Diego, CA). A p-value of <0.05 was used as significant in all cases.
